# Supplementary material for: HIVprotI: an integrated web based platform for prediction and design of HIV proteins inhibitors
Source: J Cheminform. 2018 Mar 9;10:12. doi: 10.1186/s13321-018-0266-y (PMC5845081; doi:10.1186/s13321-018-0266-y)
Supplement: Supplementary file 2 — Additional file 2. Source code of HIVProtI web server. [file 13321_2018_266_MOESM2_ESM.zip › HIVprotI_Source-code/hivprotI/weka-3-6-6/doc/weka/clusterers/AbstractClusterer.html]

AbstractClusterer


---


|  |  |  |
| --- | --- | --- |
| **Overview** \| **Package** \| **Class** \| **Tree** \| **Deprecated** \| **Index** \| **Help** \| **Weka's home** | |  |
| PREV CLASS   **NEXT CLASS** | **FRAMES**    **NO FRAMES**     **All Classes** |  |
| SUMMARY: NESTED \| FIELD \| CONSTR \| METHOD | DETAIL: FIELD \| CONSTR \| METHOD |  |


---


## weka.clusterers Class AbstractClusterer

```
java.lang.Object
  weka.clusterers.AbstractClusterer
```

**All Implemented Interfaces:**: java.io.Serializable, java.lang.Cloneable, Clusterer, CapabilitiesHandler, RevisionHandler

**Direct Known Subclasses:**: AbstractDensityBasedClusterer, CLOPE, DBScan, HierarchicalClusterer, OPTICS, RandomizableClusterer, RandomizableSingleClustererEnhancer, SingleClustererEnhancer

---

``` public abstract class AbstractClusterer extends java.lang.Object implements Clusterer, java.lang.Cloneable, java.io.Serializable, CapabilitiesHandler, RevisionHandler ```

Abstract clusterer.

**Version:**
:   $Revision: 5537 $

**Author:**
:   Mark Hall (mhall@cs.waikato.ac.nz)

**See Also:**: Serialized Form

---

**Constructor Summary**

`AbstractClusterer()`


| **Method Summary** | |
| --- | --- |
| `abstract  void` | `buildClusterer(Instances data)`             Generates a clusterer. |
| `int` | `clusterInstance(Instance instance)`             Classifies a given instance. |
| `double[]` | `distributionForInstance(Instance instance)`             Predicts the cluster memberships for a given instance. |
| `static Clusterer` | `forName(java.lang.String clustererName, java.lang.String[] options)`             Creates a new instance of a clusterer given it's class name and (optional) arguments to pass to it's setOptions method. |
| `Capabilities` | `getCapabilities()`             Returns the Capabilities of this clusterer. |
| `java.lang.String` | `getRevision()`             Returns the revision string. |
| `static Clusterer[]` | `makeCopies(Clusterer model, int num)`             Creates copies of the current clusterer. |
| `static Clusterer` | `makeCopy(Clusterer model)`             Creates a deep copy of the given clusterer using serialization. |
| `abstract  int` | `numberOfClusters()`             Returns the number of clusters. |

**Methods inherited from class java.lang.Object**

`equals, getClass, hashCode, notify, notifyAll, toString, wait, wait, wait`

**Constructor Detail**

### AbstractClusterer

```
public AbstractClusterer()
```


**Method Detail**

### buildClusterer

```
public abstract void buildClusterer(Instances data)
                             throws java.lang.Exception
```

:   Generates a clusterer. Has to initialize all fields of the clusterer
    that are not being set via options.

    :   **Specified by:**: `buildClusterer` in interface `Clusterer`
    :   **Parameters:**: `data` - set of instances serving as training data **Throws:**: `java.lang.Exception` - if the clusterer has not been generated successfully

---


### clusterInstance

```
public int clusterInstance(Instance instance)
                    throws java.lang.Exception
```

:   Classifies a given instance. Either this or distributionForInstance()
    needs to be implemented by subclasses.

    :   **Specified by:**: `clusterInstance` in interface `Clusterer`
    :   **Parameters:**: `instance` - the instance to be assigned to a cluster **Returns:**: the number of the assigned cluster as an integer **Throws:**: `java.lang.Exception` - if instance could not be clustered successfully

---


### distributionForInstance

```
public double[] distributionForInstance(Instance instance)
                                 throws java.lang.Exception
```

:   Predicts the cluster memberships for a given instance. Either
    this or clusterInstance() needs to be implemented by subclasses.

    :   **Specified by:**: `distributionForInstance` in interface `Clusterer`
    :   **Parameters:**: `instance` - the instance to be assigned a cluster. **Returns:**: an array containing the estimated membership probabilities of the test instance in each cluster (this should sum to at most 1) **Throws:**: `java.lang.Exception` - if distribution could not be computed successfully

---


### numberOfClusters

```
public abstract int numberOfClusters()
                              throws java.lang.Exception
```

:   Returns the number of clusters.

    :   **Specified by:**: `numberOfClusters` in interface `Clusterer`
    :   **Returns:**: the number of clusters generated for a training dataset. **Throws:**: `java.lang.Exception` - if number of clusters could not be returned successfully

---


### forName

```
public static Clusterer forName(java.lang.String clustererName,
                                java.lang.String[] options)
                         throws java.lang.Exception
```

:   Creates a new instance of a clusterer given it's class name and
    (optional) arguments to pass to it's setOptions method. If the
    clusterer implements OptionHandler and the options parameter is
    non-null, the clusterer will have it's options set.

    :   **Parameters:**: `clustererName` - the fully qualified class name of the clusterer: `options` - an array of options suitable for passing to setOptions. May be null. **Returns:**: the newly created search object, ready for use. **Throws:**: `java.lang.Exception` - if the clusterer class name is invalid, or the options supplied are not acceptable to the clusterer.

---


### makeCopy

```
public static Clusterer makeCopy(Clusterer model)
                          throws java.lang.Exception
```

:   Creates a deep copy of the given clusterer using serialization.

    :   **Parameters:**: `model` - the clusterer to copy **Returns:**: a deep copy of the clusterer **Throws:**: `java.lang.Exception` - if an error occurs

---


### makeCopies

```
public static Clusterer[] makeCopies(Clusterer model,
                                     int num)
                              throws java.lang.Exception
```

:   Creates copies of the current clusterer. Note that this method
    now uses Serialization to perform a deep copy, so the Clusterer
    object must be fully Serializable. Any currently built model will
    now be copied as well.

    :   **Parameters:**: `model` - an example clusterer to copy: `num` - the number of clusterer copies to create. **Returns:**: an array of clusterers. **Throws:**: `java.lang.Exception` - if an error occurs

---


### getCapabilities

```
public Capabilities getCapabilities()
```

:   Returns the Capabilities of this clusterer. Derived classifiers have to
    override this method to enable capabilities.

    :   **Specified by:**: `getCapabilities` in interface `Clusterer` **Specified by:**: `getCapabilities` in interface `CapabilitiesHandler`
    :   **Returns:**: the capabilities of this object **See Also:**: `Capabilities`

---


### getRevision

```
public java.lang.String getRevision()
```

:   Returns the revision string.

    :   **Specified by:**: `getRevision` in interface `RevisionHandler`
    :   **Returns:**: the revision


---


|  |  |  |
| --- | --- | --- |
| **Overview** \| **Package** \| **Class** \| **Tree** \| **Deprecated** \| **Index** \| **Help** \| **Weka's home** | |  |
| PREV CLASS   **NEXT CLASS** | **FRAMES**    **NO FRAMES**     **All Classes** |  |
| SUMMARY: NESTED \| FIELD \| CONSTR \| METHOD | DETAIL: FIELD \| CONSTR \| METHOD |  |


---
